# Six Express Sequence Tag–Simple Sequence Repeat Primers Reveal Genetic Diversity in the Cultivars of Three *Zanthoxylum* Species

**DOI:** 10.3390/cimb45090454

**Published:** 2023-08-30

**Authors:** Yangchuan Deng, Zhoujian He, Yanlin Li, Meng Ye, Li Xiang

**Affiliations:** 1 College of Forestry, Sichuan Agricultural University, Huimin Road 211, Wenjiang District, Chengdu 611130, China; yangchuandeng118@163.com (Y.D.); hezhouj@163.com (Z.H.); lyl1371670692@163.com (Y.L.); 2 Institute of Chinese Materia Medica, China Academy of Chinese Medical Sciences, No. 16 Nanxiaojie, Dongzhimennei Ave., Beijing 100700, China

**Keywords:** *Zanthoxylum*, cultivar, genetic diversity, EST-SSR, population

## Abstract

*Zanthoxylum* (Sichuan pepper), with its rich cultivars, has long been widely cultivated in China for its unique seasoning and medicinal uses, but most of its cultivars have similar morphological characteristics. Therefore, we hypothesized that the genetic diversity of *Zanthoxylum* cultivars is low because of their apomixis and long cultivation history. In this study, we aimed to investigate the genetic diversity of three *Zanthoxylum* species on the cultivar level based on express sequence tag–simple sequence repeat (EST-SSR) primers. In total, 121 samples of three *Zanthoxylum* species (*Z. bungeanum, Z. armatum* and *Z. piperitum*) were collected from different areas in China for genetic diversity analysis. A total of six specificity and polymorphism EST-SSR primers, which we selected from among 120 primers based on two transcriptomes (*Z. bungeanum, Z. armatum*) in our earlier study, were used to evaluate genetic diversity based on capillary electrophoresis technology. The results of our analysis using the unweighted pair group method with arithmetic mean (UPGMA) indicated that most of the samples are clustered in one clade in the UPGMA dendrogram, and the average genetic distance was 0.6409. Principal component analysis (PCA) showed that *Z. piperitum* may have a closer genetic relationship with *Z. bungeanum* than with *Z. armatum*. An analysis of molecular variation (AMOVA) showed that the genetic variation mainly stemmed from individuals within populations; the genetic differentiation coefficient (PhiPT) was 0.429, the gene flow (*Nm*) between populations was 0.333, and the differences among populations were not significant (*p* > 0.001). For the intraspecific populations of ZB, the percentage of genetic variation was 53% among populations and 47% within populations, with non-significant differences between populations (*p* > 0.001). The genetic differentiation coefficient (PhiT) was 0.529, and the gene flow (*Nm*) was 0.223. For the intraspecific populations of ZA, the results indicated that the percentage of genetic variation was 29% among populations and 71% within populations, with non-significant differences between populations (*p* > 0.001); the genetic differentiation coefficient (PhiPT) was 0.293, and the gene flow (*Nm*) was 0.223. Through genetic structure analysis (GSA), we predicted that these 121 samples belonged to two optimal subgroups, which means that all the samples probably originated from two gene pools. Above all, this indicated that the genetic diversity of the 121 *Zanthoxylum* samples was relatively low at both the species and cultivar levels, a finding which was consistent with our initial assumptions. This study provides a reference, with molecular-level data, for the further identification of *Zanthoxylum* species.

## 1. Introduction

Sichuan pepper belongs to the genus *Zanthoxylum* (family Rutaceae), which includes a wide variety of shrubs and trees. To date, more than 250 species of *Zanthoxylum* have been identified, most of which are distributed in tropical and subtropical Oceania, Africa, and Asia [1,2,3]. In China, 41 species belonging to the genus *Zanthoxylum* have been identified, 25 of which are endemic [4]. In addition, several major species have been cultivated for their aromatic, edible, and medicinal properties [5,6,7,8]. Cultivars of *Zanthoxylum* species are currently being cultivated in China due to their economic and scientific value. Among these species, *Zanthoxylum bungeanum* Maxim. and *Zanthoxylum armatum* DC. are the main species of Sichuan pepper in mainland China [9]. However, an increasing number of cultivars of *Z. bungeanum* and *Z. armatum* are being domesticated in China. Some species and cultivars can be easily confused because of their similar morphological characteristics [10]. For instance, “Qinghuajiao” in trade is often regarded as *Z. schinifolium,* but according to the research, the majority of “Qinghuajiao” products are mostly *Z. armatum* [9,11]. Likewise, “Tengjiao”, “Jinyang Qinghuajiao” and “Jiuye Qinghuajiao” are all cultivars of *Z. armatum*, although they are generally treated as different species [12]. For most of the plants, their isolated geographical environments and different climates usually lead to consolidated differences between taxa. However, most *Zanthoxylum* species exhibit apomixis, in which differences between cultivars may be negligible. Therefore, we hypothesized that the genetic diversity of *Zanthoxylum* species is low on the cultivar level because of their apomixis and stable genotypes, although they have many different names and are distributed in several different areas [13,14].

Unfortunately, the published literature on the genetic diversity of *Zanthoxylum* species and the relationship between their morphological traits and genetic diversity is lacking. The existing studies have mainly focused on traditional uses such as medicine [15,16,17], phytochemical constituents [18,19,20] and antioxidant activities [21,22,23]. However, there are few studies on genetic diversity. Medhi et al. (2014) utilized random amplified polymorphic DNA (RAPD) and inter-simple sequence repeat (ISSR) analysis to explore the genetic diversity of three *Zanthoxylum* species, and the results revealed the presence of a significant variability within and between the different *Zanthoxylum* species [24]. Sun et al. (2010) investigated *Z. schinifolium* ecotypes using the common primers of the ITS region [25]. Feng et al. (2020) investigated the genetic diversity, population structure and evolutionary history of *Zanthoxylum*, and the results indicated that the *Z. bungeanum* found in China most likely originated from southeastern Gansu Province [26]. Kim et al. (2017) developed microsatellite markers as tools for the investigation of *Z. schinifolium*. They found 15 polymorphic and 3 monomorphic microsatellite markers for the genetic study of *Z. schinifolium*. Furthermore, 3 of the 11 cross-amplified primers could distinguish between two *Zanthoxylum* species [27]. Su et al. (2022) investigated the developmental mechanism of *Z. bungeanum* prickles via morphological and multiomic analyses. The results showed that nine differentially expressed genes related to prickle development were screened and validated [28]. Hence, it is highly important to develop a reliable method for detecting the genetic diversity of *Zanthoxylum* species and cultivars.

The EST-SSR marker with conserved sequences has been utilized to design primers with a combination of SSR and transcriptome techniques. This method can be used to conveniently and rapidly screen samples by relying on more accurate and specific primers for genetic diversity, classification and fingerprinting [29]. Moreover, this approach has previously been used for various species, such as wheat [30], grape [31], rice [32], soybean [33] and orange [34]. Such studies on *Zanthoxyum* have also been reported, in which similar techniques based on the transcriptome were applied, but with different aims [35,36,37]. Therefore, we sequenced transcriptomes of both *Z. bungeanum* and *Z. armatum*, hoping to screen EST-SSR primers for a genetic diversity analysis of the *Zanthoxylum* cultivars examined in our previously published article [38]. A total of 36.76 G high-quality clean data were screened for the subsequent analysis, while 64,944 and 75,669 unigenes were obtained from *Z. bungeanum* and *Z. armatum*, respectively. In total, 12,746 SSR loci were identified in 10,595 unigenes of *Z. bungeanum*, and 15,096 SSR loci were identified in 12,612 unigenes of *Z. armatum*. A total of 60 pairs of EST-SSR primers were randomly selected from both *Z. armatum* and *Z. bungeanum*, respectively. Six pairs showed significant specificity and polymorphism in 12 samples [38]. In this study, these six selected EST-SSR primers were used to investigate the genetic diversity of 121 *Zanthoxylum* cultivars based on the UPGMA, PCA, AMOVA and GSA. We aimed to reveal the genetic diversity among these *Zanthoxylum* cultivars through the EST-SSR method.

## 2. Materials and Methods

### 2.1. Materials

**Figure 1:**
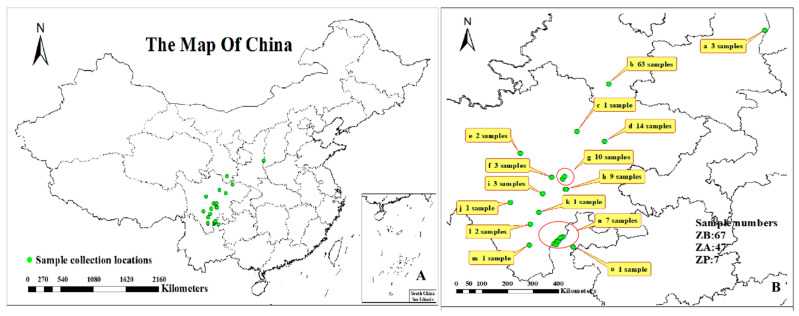
Map showing the sites from which 121 *Zanthoxylum* samples were collected for genetic diversity analysis. (**A**): Distribution of sampling sites throughout China. Scale bar = 0–2160 km. (**B**): Distribution of sampling points in locally zoomed maps. Scale bar = 0–400 km. ZB (67), ZA (47), ZP (7). ZB: *Zanthoxylum bungeanum.* ZA: *Zanthoxylum armatum.* ZP: *Zanthoxylum piperitum.* Green pot: sample collection location. **a**: Three samples, *Z. bungeanum* (cultivars, accession: 9–11). **b:** Sixty-three samples, *Z. bungeanum*: (56 cultivars, accession: 12–67); *Z. piperitum* (7 cultivars, accession: 115–121). **c**: One sample, *Z. bungeanum* (cultivar, accession: 8). **d**: Fourteen samples, *Z. armatum* (14 cultivars and 3 wild-type, accession: 68–81). **e**: Two samples, *Z. bungeanum* (cultivars, accession: 4–5). **f**: Three samples, *Z. armatum* (wild-type, accession: 82–84). **g**: Ten samples, *Z. armatum* (cultivars, accession: 86–88, 98–104). **h**: Nine samples, *Z. armatum* (cultivars, accession: 89–97). **i**: Three samples, *Z. bungeanum* (2 cultivars, accession: 6–7); *Z. armatum* (1 cultivar, accession: 85). **j:** One sample, *Z. bungeanum* (cultivar, accession: 1). **k**: 1 sample, *Z. bungeanum* (cultivar, accession: 2). **l**: Two samples, *Z. bungeanum* (1 cultivar, accession: 3); *Z. armatum* (1 cultivar, accession: 113). **m**: One sample, *Z. armatum* (cultivar, accession: 112). **n**: Seven samples, *Z. armatum* (cultivars, accession: 105–111). **o**: One sample, *Z. armatum* (cultivar, accession: 114). All sample information is shown in Supplementary Table S1.

The 121 *Zanthoxylum* samples (dried pericarp or fresh leaves), which were collected from different provinces in China from 2015 to 2017, were tested for genetic diversity (Figure 1). These 121 samples included 67 *Z. bungeanum* (ZB) samples, 47 *Z. armatum* (ZA) samples and 7 *Zanthoxylum piperitum* De Candolle (ZP) samples [39] (Supplementary Table S1). All samples were authenticated by Professor Meng Ye, Professor of College of Forestry, Sichuan Agricultural University. In particular, samples ZB-HCDHP (Irr)-1, ZB-HCDHP (Irr)-2, and ZB-HCDHP (Irr)-3 (Accession: 9–11) were irradiated; sample ZB-X (MF) (Accession: 67) only had male flowers. All samples were selected at random with three replicates, frozen in liquid nitrogen and stored at −80 °C after being returned to the laboratory.

### 2.2. DNA Isolation, PCR Amplification and Capillary Electrophoresis Fluorescence

Tissues (100 mg) of the 121 *Zanthoxylum* samples were used to extract DNA in accordance with the protocol of the DNA Secure Plant Kit (Tiangen Biotech (Beijing) Co., Ltd., Beijing, China, DP320-02). The DNA was tested via 1% agarose gel electrophoresis under a voltage of 120 V for 15 min.

The 20 µL reaction mixtures used for PCR amplification (Bio-Rad C1000 Touch, Hercules, CA, USA) comprised the following components: 14.8 µL of ddH_2_O, 0.4 µL of dNTPs, 2 µL of buffer, 0.3 µL of forward primer (20 µM), 0.3 µL of reverse primer (20 µM), 0.2 µL of DNA and 0.2 µL of Taq polymerase. The PCR program used was as follows: predenaturation at 94 °C for 5 min; 35 cycles of denaturation at 94 °C for 30 s, renaturation at 55 °C for 35 s and extension at 72 °C for 40 s; and a final extension at 72 °C for 3 min, followed by storage at 4 °C.

After mixing the formamide with the molecular weight internal standard at a volume ratio of 100:1, 9 μL was taken and added to the upper sample plate, and then 1 μL of 10-fold-diluted PCR product was added. Then, capillary electrophoresis fluorescence was carried out using an ABI PRISM 3730XL sequencer.

### 2.3. Polymorphism Assessment of Primers

The polymorphism of the six pairs of primers, including Nei’s gene diversity (*h*) [40], Shannon’s information index (*I*) [41] and the percentage of polymorphic loci, was determined using POPGENE32 VERSION1.31 software. Nei’s standard genetic distance was determined using the following formula [40]:

$$\begin{array}{l} Jx=\sum_{i=1}^{r}\sum_{j=1}^{k}X_{ij}^{2}/n,\;\\ Jy=\sum_{i=1}^{r}\sum_{j=1}^{k}Y_{ij}^{2}/n,\;\\ Jyx=\sum_{i=1}^{r}\sum_{j=1}^{k}X_{ij}Y_{ij}/n \end{array}$$

$X_{ij}$, $Y_{ij}$: gene frequency of the *k*-th allele on locus *r* in populations *X* and *Y*.

### 2.4. Genetic Distance Calculation, UPGMA and PCA

The raw data obtained using the ABI PRISM 3730XL sequencer were analyzed using Fragment (Plant) analysis VERSION 1.81 software in Gene Marker 2.2, and the fragment sizes obtained were analyzed by comparing and analyzing the position of the molecular weight internal standard in each lane with the position of the peak of each sample.

After the fragment sizes were confirmed, all the fragments were converted into a matrix of “0” and “1”, representing no production and substantial production, respectively. Based on the matrix of “0” and “1”, the genetic distances were calculated using NTSYSpc 2.11 software. The unweighted pair group method with arithmetic mean analysis (UPGMA) was performed using MEGA7.0 software. Principal component analysis (PCA) was performed using Canoco 4.5 software.

### 2.5. Analysis of Molecular Variance (AMOVA) and Genetic Structure Analysis (GSA)

Based on the fragment sizes of the PCR products, GenAlex 6.5 software [42] was used to perform an analysis of molecular variance (AMOVA) in order to calculate the genetic variation among the species and the coefficient of genetic differentiation (PhiPT), thus revealing the genetic component of the population. For the entire individual genetic structure analysis (GSA), a genetic structure calculation was performed using STRUCTURE VERSION 2.3.4. based on the Bayesian algorithm method [43,44]. At the beginning of the procedure, the number of Markov chain Monte Carlo (MCMC) iterations was set to 100,000 based on the admixture–mixture model, while it was set to 100,000 iterations during the burnin period and then 500,000 after the burnin period. Furthermore, the K-value was set at 1–3, and each K-value iteration was run 10 times. The STRUCTURE results were further processed using the Structure Harvester online tool and CLUMMP VERSION 1.1.2 software [45], and the final results were calculated and graphically presented using EXCEL 2019.

## 3. Results

### 3.1. Primers Showed Excellent Specificity and Polymorphism

In our previous study, six primer pairs showed significant specificity and polymorphism in 12 samples. Therefore, the same six pairs of EST-SSR primers were used in this trial to analyze 121 samples of *Zanthoxylum* species based on capillary electrophoresis technology. Two pairs of primers originated from *Z. armatum*, whereas the other four were derived from *Z. bungeanum* (Supplementary Table S2). We succeeded in amplifying 51 allelic loci, with a mean number of 8.5 polymorphic loci, using these primer pairs. The average number of amplified allelic genes (*Na*) was two, the average number of effective alleles (*Ne*) was 1.3323, the average Nei’s genetic diversity (*h*) was 0.2069, and the Shannon information index (*I*) was 0.3273. These values clearly indicated that the EST-SSR primers derived from the *Zanthoxylum* species themselves showed excellent specificity and polymorphism for the 121 samples used in this study. Among these primers, primer ZB26 (SSR sequence: 5′-(GGA)_5_(GGT)_5_)-3′) showed better characteristics. Specifically, its number of amplified allelic genes (*Na*) was two, its number of effective alleles (*Ne*) was 1.2738, the Nei’s genetic diversity (*h*) was 0.1608, the Shannon information index (*I*) was 0.2543, and the number of polymorphic loci was 13. Furthermore, polymorphisms of six primers for every single species were investigated. Specifically, for *Z. bungeanum*, the mean number of polymorphic loci was 6.8, the average number of amplified allelic genes (*Na*) was 1.8338, the average number of effective alleles (*Ne*) was 1.3280, the average Nei’s genetic diversity (*h*) was 0.1980, and the Shannon information index (*I*) was 0.3062. For *Z. armatum*, the mean number of polymorphic loci was 4.8, the average number of amplified allelic genes (*Na*) was 1.5733, the average number of effective alleles (*Ne*) was 1.2147, the average Nei’s genetic diversity (*h*) was 0.1305, and the Shannon information index (*I*) was 0.2059. For *Z. piperitum*, the average number of polymorphic loci was 3.7, the average number of amplified allelic genes (*Na*) was 1.3210, the average number of effective alleles (*Ne*) was 1.1630, the average Nei’s genetic diversity (*h*) was 0.0954, and the Shannon information index (*I*) was 0.1466.

### 3.2. Most Samples Are Clustered in Correct Clade in the UPGMA Dendrogram

**Figure 2:**
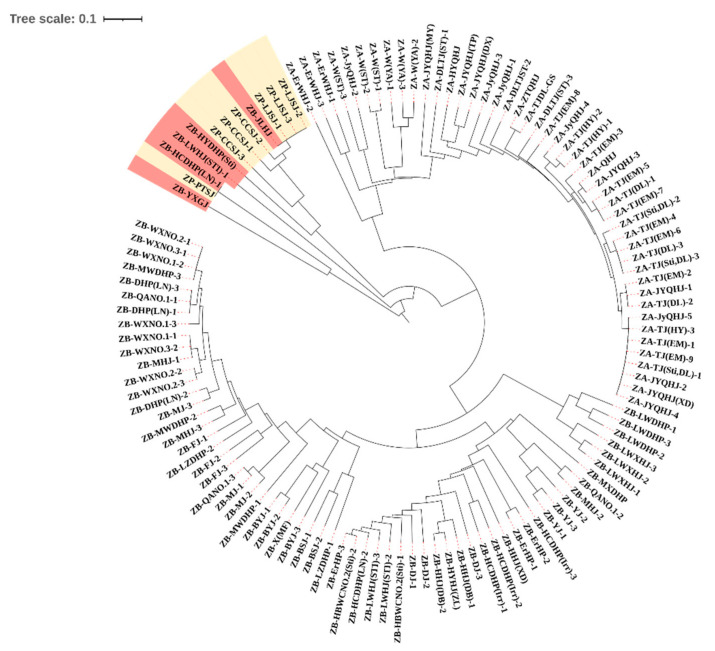
*Zanthoxylum* clustering dendrogram of 121 samples based on the genetic distance and the UPGMA method using NTSYSpc 2.11 software and MEGA7.0 (tree scale: 0.1). ZB: *Zanthoxylum bungeanum.* ZA: *Zanthoxylum armatum.* ZP: *Zanthoxylum piperitum*. Red fonts: ZB samples in the clade. Yellow fonts: ZP samples in the clade.

As shown in Figure 2, on the basis of genetic distances and the UPGMA method, six pairs of primers were successfully used to cluster three species of the *Zanthoxylum* genus in this study, which also indicated that the EST-SSR primers originating from the *Zanthoxylum* genus themselves could distinguish between the *Zanthoxylum* species more clearly. In terms of clustering branches, the three species ZB, ZA and ZP were generally distinguished. Specifically, the whole samples were divided into three branches: one branch contained mostly ZB samples, one branch included all the ZA samples, and the final branch included all the ZP samples and coupled ZB samples [ZB-JLHJ, ZBYXGJ, ZBHYDHP(Sti), ZB-LWHJ(Sti) and ZB-HCDHP(LN)-1]. The genetic distances of the 121 samples used in this study ranged from 0 to 2.3811, with a mean of 0.6409. From an interspecies perspective, the average genetic distance between ZB and ZA was 0.7856, that between ZB and ZP was 0.8147, and that between ZA and ZP was 1.8888. Within a species, the average genetic distance was 0.5104 for all the ZB samples, 0.2333 for all the ZA samples, and 0.6481 for all the ZP samples. The genetic distances of some samples were even 0, especially for the ZA-TJ series. These data indicated that the genetic diversity of the *Zanthoxylum* samples in this study was not high, and the genetic differentiation of the intraspecific cultivars was relatively low, indicating that EST-SSR did not significantly discriminate between cultivars. All the ZP samples and several ZB samples (ZB-YXGJ, ZB-HCDHP(LN), ZB-LWHJ(STI)-1, ZB-HYDHP(STi) and ZB-(JLHJ)) were clustered close together, even within one branch, and the average genetic distance between the ZB and ZP samples was 0.8147, whereas that between the ZA and ZP samples was 1.1888. This indicated that ZP and ZB may have closer kinship with each other than with ZA, respectively.

### 3.3. The Distribution Areas of ZA Were Separated while ZP and ZB Were Well-Overlapped Based on PCA

**Figure 3:**
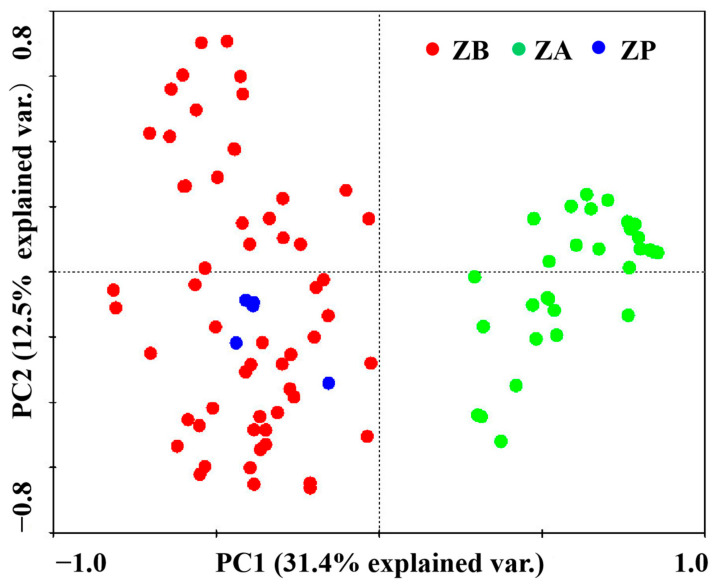
Principal component analysis of 121 *Zanthoxylum* samples. ZB: *Zanthoxylum bungeanum.* ZA: *Zanthoxylum armatum.* ZP: *Zanthoxylum piperitum.* PC1: Principal component 1. PC2: Principal component 2.

As shown in Figure 3, the results revealed that the PCA method could clearly be used to investigate the relationships of ZA with ZB and ZP species. The distribution areas of ZP and ZB were relatively well-overlapped, suggesting that ZP and ZB may have a closer genetic relationship similar to that indicated by the results of the dendrogram. On the one hand, the ZB samples were more dispersed, and the ZA samples were more compactly distributed. On the other hand, the samples of both ZB and ZA were concentrated, and some samples overlapped, indicating that the intraspecific genetic diversity of both ZB and ZA was not high.

### 3.4. The Interspecific and Intraspecific Gene Flows Were Low

As shown in Table 1, the results of the AMOVA showed that the genetic variation among and within all the populations of *Zanthoxylum* in this study was 43% and 57%, respectively, and the differences among populations were not significant (*p* > 0.001). Furthermore, the genetic differentiation coefficient (PhiPT) was 0.429, and the gene flow between populations (*Nm*) was 0.333. For the intraspecific populations of ZB, the AMOVA showed that the percentage of genetic variation was 53% among populations and 47% within populations, with non-significant differences between populations (*p* > 0.001). The genetic differentiation coefficient (PhiT) was 0.529, and the gene flow (*Nm*) was 0.223. For the intraspecific populations of ZA, the results indicated that the percentage of genetic variation was 29% among populations and 71% within populations, with non-significant differences between populations (*p* > 0.001), that the genetic differentiation coefficient (PhiPT) was 0.293, and the gene flow (*Nm*) was 0.223. The percentage of genetic variation within populations was 71%, the difference between populations was highly insignificant (*p* > 0.001), the genetic differentiation coefficient (PhiPT) was 0.293, and the gene flow (*Nm*) was 0.605. Overall, the genetic variation of the *Zanthoxylum* populations in this study originated mainly within populations rather than among populations, and the interspecific gene flows and intraspecific gene flows were not high.

### 3.5. Three Zanthoxylum Species Divided into Two Genetic Structures

**Figure 4:**
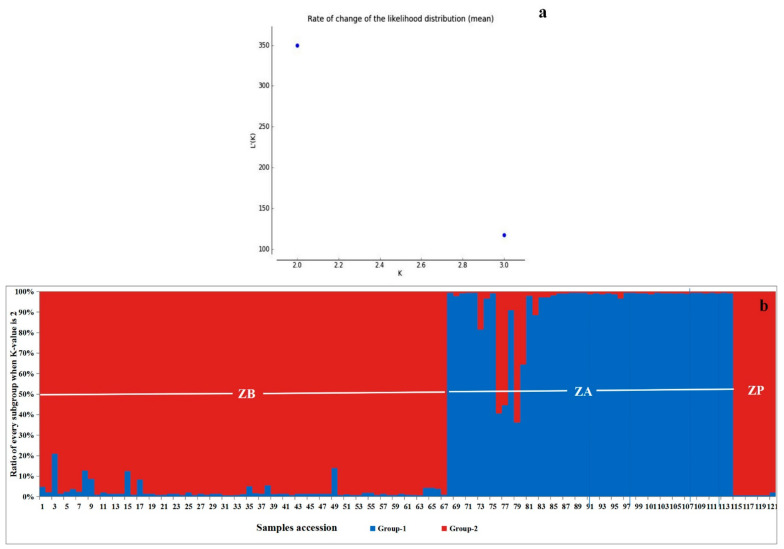
Genetic structure and K-value analysis of all samples. (**a**) Reasonable number of subgroups for all samples according to K-value analysis. (**b**) Genetic structure analysis of all *Zanthoxylum* samples in this study. ZB: *Zanthoxylum bungeanum*. ZA: *Zanthoxylum armatum*. ZP: *Zanthoxylum piperitum*.

As shown in Figure 4, the GSA indicated that the optimal population of these 121 samples could be divided into two subgroups (the K-value was 2) (Figure 4a) and could more clearly distinguish between ZB, ZA and ZP. There was a greater similarity between ZP and ZB, which aligned with the results of the clustering analysis and PCA. In addition, some samples of ZB exhibited a degree of difference based on the GSA results, showing that the genetic diversity of some samples of ZB was higher than that of ZA. This may have been due to a higher number of samples and the distribution of sampling sites for ZB. In comparison, the similarity of the ZA samples was higher. There was less genetic differentiation within species, and the genetic diversity was lower. Among the seven ZP samples, the overall degree of difference was not significant, with only the ZP-PTSJ sample being slightly different from the other six samples. The other six samples consisted of three cultivars, with one sample of ZP-PTSJ, three samples of ZP-CCSJ and three samples of ZP-JLSJ. It is possible that the one sample of ZP-PTSJ differs from the three samples of the other two cultivars, but the presence of only one sample indicated that the findings could simply be due to an experimental error.

## 4. Discussion

### 4.1. EST-SSR Validation

Different kinds of molecular markers are now universally used to analyze genetic diversity for many species rapidly and efficiently. Examples include SSR in grape [46,47], chickpea [48], and *Prunus armeniaca* [49]; RAPD in grape [46], *Apiaceae* [50] and *Piperaceae* [51]; And CAPS in *Citrus* [52], cotton [53] and *Camellia sinensis* [54]. Although the species of *Zanthoxylum* are economically important trees in China [55], relatively few studies have focused on their genetic diversity [56]. In our published study, we sequenced the transcriptomes of *Z. bungeanum* and *Z. armatum* using the Illumina-Hiseq sequencing platform, which provided a good basis for the screening of EST-SSR markers and the detection of genetic diversity [38]. The benefit of obtaining EST-SSR primers using RNA-seq is that these markers have better specificity and polymorphism compared to conventional primers, because EST-SSR is derived from cDNA and is conserved [57]. These properties are considered ideal properties for molecular markers; thus, it is best to use EST-SSR for classification and identification. However, to date, only a few studies have detected the transcriptome of *Zanthoxylum*. Zhao et al. [58] reported the results of a germination analysis of *Z. piperitum* based on its transcriptome. Meanwhile, Feng et al. [37] employed a de novo transcriptome-developed SSR of *Z. bungeanum*. In addition, Fei et al. [14] investigated the mechanism of the apomixis of *Z. bungeanum* using an miRNA-based technique. However, the majority of previous studies only focused on one species, with no comparisons being performed. On the interspecies level, numerous published studies have proved that EST-SSRs have shown accurate and clear results with respect to the identity and genetic diversity of crop plants, such as *Triticum* [59], *Hordeum vulgare* [60] and *Citrus* [61]. On the cultivar level, this technique is more likely to fail to yield distinct results, as in the case of *Zanthoxylum* [62]. In this study, the six EST-SSR markers derived from two *Zanthoxylum* species showed significant availability and polymorphism for the three *Zanthoxylum* species samples, though it seemed that they were not able to definitively identify cultivars of *Zanthoxylum* clearly, suggesting that this technique may be more appropriate for species-level identification than cultivar-level identification. Additionally, the polymorphism for *Z. piperitum* was not as good as that for *Z. bungeanum* and *Z. armatum*. One possible reason is that there were no EST-SSR primers from *Z. piperitum*; another is that the number of samples was probably too low.

### 4.2. Genetic Diversity Assessments

In the present study, 121 samples were shown to belong to three species of the *Zanthoxylum* genus. Among the different analytical methods used in this study, the K-value analysis of the genetic structure, in particular, showed that the most suitable subgroup number for these samples was two. This indicated that the genetic diversity of the *Zanthoxylum* genus is not high. This finding may be attributed to several reasons. First, although the total number of samples in this study was large, many of the samples were cultivars. Second, only six primer pairs were used in this study, and while it was possible to clearly identify three *Zanthoxylum* species, the identification of intraspecific cultivars was relatively difficult, which indicated that the ability of the EST-SSR primers to identify intraspecific cultivars was somewhat limited. Third, the genus *Zanthoxylum* has a mainly reproductive mode of apomixis and thus has a stable phenotype [13,14]. Above all, the genetic differentiations were minimal, and the phenotypes were stable.

Additionally, we compared the morphological characteristics of *Z. bungeanum, Z. armatum* and *Z. piperitum* based on previously reported research (Supplementary Table S3) [9,63,64]. Z. *piperitum* is a special and stingless pepper that comes from Japan, which can be clearly differentiated from other Sichuan peppers [63,64]. There are few articles about *Z. piperitum* species regarding morphology and molecular biology, and there are no available EST databases. In this study, the ESR-SST primers for *Z. bungeanum* and *Z. armatum* were used to analyze the genetic diversity of *Z. piperitum.* The results indicated that *Z. piperitum* may be closer to *Z. bungeanum* than to *Z. armatum*. However, the lack of appropriate primers and the sample size may have had a certain impact on the results for *Z. piperitum*, and we will make improvements in future research.

Sample ZB-X (MF), a sample with only male flowers, did not show any significant differences from the rest of the samples in terms of clustering, PCA or genetic structure, probably because only one sample was analyzed. Three samples of ZB-HCDHP (Irr) were irradiated, and the clustering results revealed that these three samples did not show any significant differences when compared with the other samples. In addition, there was no genetic difference between the three samples. Furthermore, the PCA and GSA results revealed that these three samples showed no distinctive results. Irradiation mutation breeding is an approach used to induce gene mutations, promote gene recombination and improve the recombination rate [65,66,67]. Neither extraterrestrial radiation nor male flower production were found to play an important role in the genetic structure of these species, for which we could not detect differences based on the EST-SSR analysis. Since there were too few of the two specimen types, no statistically significant differences were identified between the individual samples. None of the ZB-DHP series samples, which were more widely distributed in the ZB branch, were particularly concentrated, suggesting the existence of some differences in ZB-DHP on the molecular level in different regions. In Figure 2, one of the clades included all the samples of ZP and several samples from ZB. Among them, ZB-JLHJ and ZB-YXGJY are both varieties with a long history of cultivation in China. However, because they have been cultivated in mountainous areas for a long time, there was less traffic, less foreign introduction and less exchange of genetic resources, so this may be the reason why they are not classified in the same branch as other ZB samples, or it may be the result of the chance of the experiments that and the results are not informative.

The ZA-Ws were wild *Z. armatum* samples, whereas the ZA-TJs were a *Z. armatum* cultivar. The genetic distance results and dendrogram demonstrated that these two types of samples were relatively genetically remote, indicating that the wild *Z. armatum* type and the cultivars used in this study differed to some extent. Meanwhile, the clustering, PCA and GSA results revealed that all the ZA-TJ samples showed an extremely close genetic relationship; the genetic distances of some samples were almost 0, indicating the very limited genetic differentiation of the cultivar. This may be because the ZA-TJ samples collected in this study were derived from Sichuan Province, where the sampling was performed within a small area. Thus, based on the genetic distance results, *Zanthoxylum’s* apomixis and the small sampling areas, it is likely that the samples may have originated from a single gene pool.

AMOVA is an important tool for studying genetic diversity among the populations of species. In this study, for the entire *Zanthoxylum* genus, interspecies variation accounted for 43% of the total variation, and intraspecies variation accounted for 57%, indicating that the population variation in the three *Zanthoxylum* samples in this study mainly originated from within the populations. Specifically, 53% of the inter-population variation was accounted for in ZB, suggesting that this variation mainly originated from among populations, which may be due to the fact that a larger number of ZB samples were collected in the present study and that the collection was performed using a wide range of regional sources. The genetic differentiation coefficient (PhiPT) and gene flow (*Nm*) are important indicators for analyzing the genetic structure of populations. According to Wright et al. (1990), if the genetic differentiation coefficient (PhiPT) ranges from 0 to 0.05, this indicates that no differentiation exists between populations; if it is 0.05–0.15, this indicates that there is moderate differentiation between populations; and if it is 0.15–0.25, this indicates high differentiation. If the PhiPT is greater than 0.25, this indicates extremely differentiated populations [68]. According to the results, it can be seen that the PhiPT was greater than 0.25, whether between the three species, within the ZB populations or within the ZA populations, which indicated that there was a great deal of genetic differentiation between the different *Zanthoxylum* species and also between the intraspecific populations in the current study. Ellstrand et al. suggested that the proportion of genetic variation among populations of asexual plants increases greatly when there are large barriers to gene flow among the populations [69]. Most of the *Zanthoxylum* species reproduce via apomixis, similar to asexual lineages; thus, it is reasonable to infer that the variation among populations is relatively large. According to Buso et al.’s research, a gene flow (*Nm*) > 1 results in a high degree of gene exchange and that of 0.250 < *Nm* < 0.999 results in a moderate degree of gene exchange [70]. None of the gene flow (*Nm*) values in this experiment were beyond 1; therefore, the degree of gene exchange was not high, probably because of the apomixis reproduction.

The *Zanthoxylum* market in China mostly consists of ZB and ZA species. Due to the different climatic conditions in China, *Z. bungeanum* is cultivated in the north and *Z. armatum* is cultivated in the south [9]. Therefore, as *Z. bungeanum*, *Z. armatum* and their cultivars are the main types of Sichuan pepper commercially grown in China, it is vital to make considerable efforts to maintain the diversity of *Zanthoxylum* with respect to characteristics such as its aroma, stinglessness and pungency.

## 5. Conclusions

In this research, we used six EST-SSR primers to detect the genetic diversity of three *Zanthoxylum* species, with 121 samples, using capillary electrophoresis technology. According to the UPGMA and PCA, the 121 samples were divided into three clades, and *Z. piperitum* and *Z. bungeanum* were more closely related to each other than either was to *Z. armatum*. On the basis of STRUCTURE, all the samples probably originated from two gene pools. Additionally, the AMOVA indicated that the differences among interspecies and intraspecies populations were not significant. Above all, the *Zanthoxylum* samples examined in this study showed relatively low genetic diversity at the cultivar level, which was consistent with our initial assumptions. However, we found that the EST-SSR primers can distinguish between the different species of *Zanthoxylum* but cannot clearly identify large numbers of cultivars; this topic requires further research. This study provides a reference, with molecular-level data, for further examination of the identity of *Zanthoxylum*.

## Figures and Tables

**Table 1:** The molecular variance (AMOVA) for both interspecies and intraspecies variations.

Source of Variation		Degrees of Freedom (df)	Statistics (SS)	Mean Square (MS)	Est. Var.	Percentage of Variance Component	*p*-Value
Among Populations (AP)	Interspecific populations of ZB, ZA and ZP	2	149.543	74.771	2.224	43%	*p* > 0.001
	Intraspecific populations of ZB	21	130.700	6.224	1.721	53%	*p* > 0.001
	Intraspecific populations of ZA	9	29.320	3.258	0.523	29%	*p* > 0.001
Within Populations (WP)	Interspecific populations of ZB, ZA and ZP	118	349.854	2.965	2.965	57%	\
	Intraspecific populations of ZB	38	58.333	1.535	1.535	47%	\
	Intraspecific populations of ZA	29	36.706	1.266	1.266	71%	\
Total	Interspecific populations of ZB, ZA and ZP	120	499.397	\	5.189	100%	\
	Intraspecific populations of ZB	59	189.033	\	3.256	100%	\
	Intraspecific populations of ZA	38	66.026	\	1.789	100%	\
Genetic differentiation coefficient (PhiPT)	Interspecific populations of ZB, ZA and ZP			0.429			
	Intraspecific populations of ZB			0.529			
	Intraspecific populations of ZA			0.293			
Gene flow (*Nm*)	Interspecific populations of ZB, ZA and ZP			0.333			
	Intraspecific populations of ZB			0.223			
	Intraspecific populations of ZA			0.605			

PhiPT = AP/(WP + AP) = AP/Total; *Nm* = [(1/PhiPT) − 1]/4; AP = Est. Var. Among Pops, WP = Est. Var. Within Pops.

## Data Availability

The data presented in this study are openly available with the article and Supplementary Materials.

## Supplementary Materials

The following supporting information can be downloaded at: https://www.mdpi.com/article/10.3390/cimb45090454/s1, Table S1: Table of 121 Zanthoxylum Samples information; Table S2: Table information of six pairs of EST-SSR primers and polymorphsim results; Table S3: Table of Morphology characteristics of *Z. bungeanum*, *Z. armatum* and *Z. piperitum*.

## Abbreviations

EST-SSR	Express sequence tag–simple sequence repeat
ZB	*Zanthoxylum bungeanum*
ZA	*Zanthoxylum armatum*
ZP	*Zanthoxylum piperitum*
RAPD	Random Amplified Polymorphic DNA
ISSR	Inter-Simple Sequence Repeat
MCMC	Markov Chain Monte Carlo
PCA	Principal Component Analysis
UPGMA	Unweighted Pair Group Method with Arithmetic Mean Analysis
GSA	Genetic Structure Analysis
NR	Non-Redundant Protein Sequence Database
COG	Clusters of Orthologous Group
KEGG	Kyoto Encyclopedia of Genes and Genomes
GO	Gene Ontology
