# Supplementary material for: Six Express Sequence Tag–Simple Sequence Repeat Primers Reveal Genetic Diversity in the Cultivars of Three Zanthoxylum Species
Source: Curr Issues Mol Biol. 2023 Aug 30;45(9):7183–96. doi: 10.3390/cimb45090454 (PMC10529843; doi:10.3390/cimb45090454)
Supplement: Supplementary file 1 [file cimb-45-00454-s001.zip › Supplementary Table S1. Table of 121 Zanthoxylum Samples information.pdf]

Supplementary Table S1. Table of 121 *Zanthoxylum* Samples information

Accession	Sample	Abbreviation	Sample origin	Time Day/Month/Year	Altitude (m)	Location	Remark	Appraiser
1	*Z.bungeanum* 'Jiulong Huajiao'	ZB-JLHJ	Sichuan Province	-	2800	28°59'N 101°30'E	-	Meng Ye
2	*Z. bungeanum* 'Yuexi Gongjiao'	ZB-YXGJ	Liangshan Yi Autonomous Prefecture Yuexi County	-	-	-	-	Meng Ye
3	*Z. bungeanum* 'Honghuajiao'(XD)	ZB-HHJ(XD)	Sichuan Province Xide County	-	1600	-	-	Meng Ye
4	*Z. bungeanum* 'Honghuajiao'(DB)-1	ZB-HHJ(DB)-1	Sichuan Province Danba County	9/12/2016	-	-	-	Meng Ye
5	*Z. bungeanum* 'Honghuajiao'(DB)-2	ZB-HHJ(DB)-2	Sichuan Province Danba County	9/12/2016	-	-	-	Meng Ye
6	*Z.bungeanum* 'Hanyuan Dahongpao' (Stingless)	ZB-HYDHP(Sti)	Sichuan Province Hanyuan County	2015	-	-	Stingless	Meng Ye
7	*Z. bungeanum* 'Hanyuan Huajiao'(Zhenglu）	ZB-HYHJ（ZL）	Sichuan Province Hanyuan County	2015	-	-	-	Meng Ye
8	*Z.bungeanum*'Maoxian Dahongpao'	ZB-MXDHP	Sichuan Province Maoxian County	-	-	-	-	Meng Ye
9	*Z.bungeanum* 'Hancheng Dahongpao' (Irradiated)-1	ZB-HCDHP(Irr)--1	Shanxi Province Hancheng City	17/11/2017	-		Irradiated	Meng Ye

10	*Z.bungeanum* ‘Hancheng Dahongpao’ (Irradiated)-2	ZB-HCDHP(Irr)-2	Shanxi Province Hancheng City	17/11/2017	-		Irradiated	Meng Ye
11	*Z.bungeanum* ‘Hancheng Dahongpao’(Irradiated)-3	ZB-HCDHP(Irr)-3	Shanxi Province Hancheng City	17/11/2017	-		Irradiated	Meng Ye
12	*Z.bungeanum* ‘Dahongpao’(LN)-1	ZB-DHP(LN)-1	Gansu Province Longnan City	30/6/2017	1314	33°27'46″N 104°57'62″	-	Meng Ye
13	*Z.bungeanum* ‘Dahongpao’(LN)-2	ZB-DHP(LN)-2	Gansu Province Longnan City	30/6/2017	1314	33°27'46″N 104°57'62″	-	Meng Ye
14	*Z.bungeanum* ‘Dahongpao’(LN)-3	ZB-DHP(LN)-3	Gansu Province Longnan City	30/6/2017	1314	33°27'46″N 104°57'62″	-	Meng Ye
15	*Z.bungeanum* ‘Hancheng Dahongpao’(LN)-1	ZB-HCDHP(LN)-1	Gansu Province Longnan City	30/6/2017	1314	33°27'46″N 104°57'62″	-	Meng Ye
16	*Z.bungeanum* ‘Hancheng Dahongpao’(LN)-2	ZB-HCDHP(LN)-2	Gansu Province Longnan City	30/6/2017	1314	33°27'46″N 104°57'62″	-	Meng Ye
17	*Z.bungeanum* ‘Maowen Dahongpao’-1	ZB-MWDHP-1	Gansu Province Longnan City	30/6/2017	1314	33°27'46″N 104°57'62″	-	Meng Ye
18	*Z.bungeanum* ‘Maowen Dahongpao’-2	ZB-MWDHP-2	Gansu Province Longnan City	30/6/2017	1314	33°27'46″N 104°57'62″	-	Meng Ye
19	*Z.bungeanum* ‘Maowen Dahongpao’-3	ZB-MWDHP-3	Gansu Province Longnan City	30/6/2017	1314	33°27'46″N 104°57'62″	-	Meng Ye
20	*Z.bungeanum* ‘Linzhou Dahongpao’-1	ZB-LZDHP-1	Gansu Province Longnan City	30/6/2017	1314	33°27'46″N 104°57'62″	-	Meng Ye

21	*Z.bungeanum* ‘Linzhou Dahongpao’-2	ZB-LZDHP-2	Gansu Province Longnan City	30/6/2017	1314	33°27'46″N 104°57'62″	-	Meng Ye
22	*Z.bungeanum* ‘Laiwu Xiaohongjiao’-1	ZB-LWXHJ-1	Gansu Province Longnan City	9/12/2016	1314	33°27'46″N 104°57'62″	-	Meng Ye
23	*Z.bungeanum* ‘Laiwu Xiaohongjiao’-2	ZB-LWXHJ-2	Gansu Province Longnan City	9/12/2016	1314	33°27'46″N 104°57'62″	-	Meng Ye
24	*Z.bungeanum* ‘Laiwu Xiaohongjiao’-3	ZB-LWXHJ-3	Gansu Province Longnan City	9/4/2017	1314	33°27'46″N 104°57'62″	-	Meng Ye
25	*Z.bungeanum* ‘Laiwu Huajiao’(stingless)-1	ZB-LWHJ(STI)-1	Gansu Province Longnan City	9/4/2017	1316	33°27'46″N 104°57'63″	stingless	Meng Ye
26	*Z.bungeanum* ‘Laiwu Huajiao’(stingless)-2	ZB-LWHJ(STI)--2	Gansu Province Longnan City	9/4/2017	1316	33°27'46″N 104°57'63″	stingless	Meng Ye
27	*Z.bungeanum* ‘Laiwu Huajiao’(stingless)-3	ZB-LWHJ(STI)--3	Gansu Province Longnan City	9/4/2017	1316	33°27'46″N 104°57'63″	stingless	Meng Ye
28	*Z.bungeanum* ‘Laiwu Dahongpao’-1	ZB-LWDHP-1	Gansu Province Longnan City	14/5/2017	1316	33°27'46″N 104°57'63″	-	Meng Ye
29	*Z.bungeanum* ‘Laiwu Dahongpao’-2	ZB-LWDHP-2	Gansu Province Longnan City	14/5/2017	1316	33°27'46″N 104°57'63″	-	Meng Ye
30	*Z.bungeanum* ‘Laiwu Dahongpao’-3	ZB-LWDHP-3	Gansu Province Longnan City	29/6/2017	1316	33°27'46″N 104°57'63″	-	Meng Ye
31	*Z.bungeanum* ‘Erhongpao’-1	ZB-ErHP-1	Gansu Province Longnan City	30/6/2017	1316	33°27'46″N 104°57'63″	-	Meng Ye
32	*Z.bungeanum* ‘Erhongpao’-2	ZB-ErHP-2	Gansu Province Longnan City	30/6/2017	1316	33°27'46″N 104°57'63″	-	Meng Ye

33	*Z.bungeanum* 'Erhongpao'-3	ZB-ErHP-3	Gansu Province Longnan City	30/6/2017	1316	33°27'46″N 104°57'63″	-	Meng Ye
34	*Z.bungeanum* 'Youjiao'-1	ZB-YJ-1	Gansu Province Longnan City	30/6/2017	1316	33°27'46″N 104°57'63″	-	Meng Ye
35	*Z.bungeanum* 'Youjiao'-2	ZB-YJ-2	Gansu Province Longnan City	30/6/2017	1316	33°27'46″N 104°57'63″	-	Meng Ye
36	*Z.bungeanum* 'Youjiao'-3	ZB-YJ-3	Gansu Province Longnan City	30/6/2017	1316	33°27'46″N 104°57'63″	-	Meng Ye
37	*Z.bungeanum* 'Meihuajiao'-1	ZB-MHJ-1	Gansu Province Longnan City	30/6/2017	1316	33°27'46″N 104°57'63″	-	Meng Ye
38	*Z.bungeanum* 'Meihuajiao'-2	ZB-MHJ-2	Gansu Province Longnan City	30/6/2017	1316	33°27'46″N 104°57'63″	-	Meng Ye
39	*Z.bungeanum* 'Meihuajiao'-3	ZB-MHJ-3	Gansu Province Longnan City	30/6/2017	1316	33°27'46″N 104°57'63″	-	Meng Ye
40	*Z.bungeanum* 'Wuxuan NO.1'-1	ZB-WXNO.1-1	Gansu Province Longnan City	30/6/2017	1316	33°27'46″N 104°57'63″	-	Meng Ye
41	*Z.bungeanum* 'Wuxuan NO.1'-2	ZB-WXNO.1-2	Gansu Province Longnan City	30/6/2017	1316	33°27'46″N 104°57'63″	-	Meng Ye
42	*Z.bungeanum* 'Wuxuan NO.1'-3	ZB-WXNO.1-3	Gansu Province Longnan City	30/6/2017	1316	33°27'46″N 104°57'63″	-	Meng Ye
43	*Z.bungeanum* 'Wuxuan NO.2'-1	ZB-WXNO.2-1	Gansu Province Longnan City	30/6/2017	1316	33°27'46″N 104°57'63″	-	Meng Ye
44	*Z.bungeanum* 'Wuxuan NO.2'-2	ZB-WXNO.2-2	Gansu Province Longnan City	30/6/2017	1316	33°27'46″N 104°57'63″	-	Meng Ye

45	*Z.bungeanum* 'Wuxuan NO.2'-3	ZB-WXNO.2-3	Gansu Province Longnan City	30/6/2017	1316	33°27'46″N 104°57'63″	-	Meng Ye
46	*Z.bungeanum* 'Wuxuan NO.3'-1	ZB-WXNO.3-1	Gansu Province Longnan City	30/6/2017	1316	33°27'46″N 104°57'63″	-	Meng Ye
47	*Z.bungeanum* 'Wuxuan NO.3'-2	ZB-WXNO.3-2	Gansu Province Longnan City	30/6/2017	1316	33°27'46″N 104°57'63″	-	Meng Ye
48	*Z. bungeanum* 'Qin An NO.1'-1	ZB-QANO.1-1	Gansu Province Longnan City	30/6/2017	1316	33°27'46″N 104°57'63″	-	Meng Ye
49	*Z. bungeanum* 'Qin An NO.1'-2	ZB-QANO.1-2	Gansu Province Longnan City	30/6/2017	1316	33°27'46″N 104°57'63″	-	Meng Ye
50	*Z. bungeanum* 'Qin An NO.1'-3	ZB-QANO.1-3	Gansu Province Longnan City	30/6/2017	1316	33°27'46″N 104°57'63″	-	Meng Ye
51	*Z.bungeanum* 'Heibei Wuci No.2' (stingless)-1	ZB-HBWCNO.2(Sti)-1	Gansu Province Longnan City	30/6/2017	1316	33°27'46″N 104°57'63″	stingless	Meng Ye
52	*Z.bungeanum* 'Heibei Wuci No.2' (stingless)-2	ZB-HBWCNO.2(Sti)-2	Gansu Province Longnan City	30/6/2017	1316	33°27'46″N 104°57'63″	stingless	Meng Ye
53	*Z.bungeanum* 'Fengjiao'-1	ZB-FJ-1	Gansu Province Longnan City	30/6/2017	1316	33°27'46″N 104°57'63″	-	Meng Ye

54	*Z.bungeanum* 'Fengjiao'-2	ZB-FJ-2	Gansu Province Longnan City	30/6/2017	1316	33°27'46″N 104°57'63″	-	Meng Ye
55	*Z.bungeanum* 'Fengjiao'-3	ZB-FJ-3	Gansu Province Longnan City	30/6/2017	1316	33°27'46″N 104°57'63″	-	Meng Ye
56	*Z.bungeanum* 'Baishajiao'-1	ZB-BSJ-1	Gansu Province Longnan City	30/6/2017	1316	33°27'46″N 104°57'63″	-	Meng Ye
57	*Z.bungeanum* 'Baishajiao'-2	ZB-BSJ-2	Gansu Province Longnan City	30/6/2017	1316	33°27'46″N 104°57'63″	-	Meng Ye
58	*Z.bungeanum* 'Mianjiao'-1	ZB-MJ-1	Gansu Province Longnan City	30/6/2017	1316	33°27'46″N 104°57'63″	-	Meng Ye
59	*Z.bungeanum* 'Mianjiao'-2	ZB-MJ-2	Gansu Province Longnan City	30/6/2017	1316	33°27'46″N 104°57'63″	-	Meng Ye
60	*Z.bungeanum* 'Mianjiao'-3	ZB-MJ-3	Gansu Province Longnan City	30/6/2017	1316	33°27'46″N 104°57'63″	-	Meng Ye
61	*Z.bungeanum* 'Doujiao'-1	ZB-DJ-1	Gansu Province Longnan City	30/6/2017	1316	33°27'46″N 104°57'63″	-	Meng Ye
62	*Z.bungeanum* 'Doujiao'-2	ZB-DJ-2	Gansu Province Longnan City	30/6/2017	1316	33°27'46″N 104°57'63″	-	Meng Ye
63	*Z.bungeanum* 'Doujiao'-3	ZB-DJ-3	Gansu Province Longnan City	30/6/2017	1316	33°27'46″N 104°57'63″	-	Meng Ye
64	*Z.bungeanum* 'Bayuejiao'-1	ZB-BYJ-1	Gansu Province Longnan City	30/6/2017	1316	33°27'46″N 104°57'63″	-	Meng Ye

65	*Z.bungeanum* ‘Bayuejiao’-2	ZB-BYJ-2	Gansu Province Longnan City	30/6/2017	1316	33°27'46″N 104°57'63″	-	Meng Ye
66	*Z.bungeanum* ‘Bayuejiao’-3	ZB-BYJ-3	Gansu Province Longnan City	30/6/2017	1316	33°27'46″N 104°57'63″	-	Meng Ye
67	*Z. bungeanum* ‘X’(Male flower only)	ZB-X(MF)	Gansu Province Longnan City	30/6/2017	1316	33°27'46″N 104°57'63″	Male flower only	Meng Ye
68	*Z.armatum* ‘Jiuye Qinghuajiao’-1	ZA-JyQHJ-1	Sichuan Province Santai County	8/12/2016	450	31°19′N 104°50′E	-	Meng Ye
69	*Z.armatum* ‘Jiuye Qinghuajiao’-2	ZA-JyQHJ-2	Sichuan Province Santai County	8/12/2016	450	31°19′N 104°50′E	-	Meng Ye
70	*Z.armatum* ‘Jiuye Qinghuajiao’-3	ZA-JyQHJ-3	Sichuan Province Santai County	8/12/2016	450	31°19′N 104°50′E	-	Meng Ye
71	*Z.armatum* ‘Jiuye Qinghuajiao’-4	ZA-JyQHJ-4	Sichuan Province Santai County	8/12/2016	450	31°19′N 104°50′E	-	Meng Ye
72	*Z.armatum* ‘Jiuye Qinghuajiao’-5	ZA-JyQHJ-5	Sichuan Province Santai County	8/12/2016	450	31°19′N 104°50′E	-	Meng Ye
73	*Z.armatum* ‘Danlingtengjiao’(ST)-1	ZA-DLTJ(ST)-1	Sichuan Province Santai County	8/12/2016	450	31°19′N 104°50′E	-	Meng Ye
74	*Z.armatum* ‘Danlingtengjiao’(ST)-2	ZA-DLTJ(ST)-2	Sichuan Province Santai County	8/12/2016	450	31°19′N 104°50′E	-	Meng Ye
75	*Z.armatum* ‘Danlingtengjiao’(ST)-3	ZA-DLTJ(ST)-3	Sichuan Province San Tai County	8/12/2016	450	31°19′N 104°50′E	-	Meng Ye
76	*Z. armatum* ‘Erwai Huajiao’-1	ZA-ErWHJ-1	Sichuan Province Santai County	8/12/2016	450	31°19′N 104°50′E	-	Meng Ye

77	*Z.armatum* ‘Erwai Huajiao’-2	ZA-ErWHJ-2	Sichuan Province Santai County	8/12/2016	450	31°19′N 104°50′E	-	Meng Ye
78	*Z.armatum* ‘Erwai Huajiao’-3	ZA-ErWHJ-3	Sichuan Province Santai County	8/12/2016	450	31°19′N 104°50′E	-	Meng Ye
79	*Z.armatum* (Wild, ST)-1	ZA-W(ST)-1	Sichuan Province Santai County	8/12/2016	450	31°19′N 104°50′E	wild	Meng Ye
80	*Z. armatum* (Wild, ST)-2	ZA-W(ST)-2	Sichuan Province Santai County	8/12/2016	450	31°19′N 104°50′E	wild	Meng Ye
81	*Z.armatum* (Wild, ST)-3	ZA-W(ST)-3	Sichuan Province Santai County	8/12/2016	450	31°19′N 104°50′E	wild	Meng Ye
82	*Z. armatum* (Wild,YA)-1	ZA-W(YA)-1	Sichuan Province Ya'an City	8/12/2016	450	29°58'N 102°59′E	wild	Meng Ye
83	*Z.armatum* (Wild,YA)-2	ZA-W(YA)-2	Sichuan Province Ya'an City	8/12/2016	450	29°58'N 102°59′E	wild	Meng Ye
84	*Z.armatum* (Wild,YA)-3	ZA-W(YA)-3	Sichuan Province Ya'an City	8/12/2016	450	29°58'N 102°59′E	wild	Meng Ye
85	*Z. armatum* ‘Hanyuan Qinghuajiao’	ZA-HYQHJ	Sichuan Province Hanyuan County	2015	-	-	-	Meng Ye
86	*Z.armatum* ‘Tengjiao’(HY)-1	ZA-TJ(HY)-1	Sichuan Province Hongya County	8/12/2016	450	29°53′N 103°21′21″E	-	Meng Ye
87	*Z.armatum* ‘Tengjiao’(HY)-2	ZA-TJ(HY)-2	Sichuan Province Hongya County	8/12/2016	450	29°53′51″N 103°21′21″E	-	Meng Ye
88	*Z.armatum* ‘Tengjiao’(HY)-3	ZA-TJ(HY)-3	Sichuan Province Hongya County	8/12/2016	450	29°53′51″N 103°21′21″E	-	Meng Ye

89	*Z.armatum* 'Tengjiao'(EM)-1	ZA-TJ(EM)-1	Sichuan Province Emeishan City	8/12/2016	450	29°30′50″N 103°27′12″E	-	Meng Ye
90	*Z.armatum* 'Tengjiao'(EM)-2	ZA-TJ(EM)-2	Sichuan Province Emeishan City	8/12/2016	475	29°30′50″N 103°27′12″E	-	Meng Ye
91	*Z.armatum* 'Tengjiao'(EM)-3	ZA-TJ(EM)-3	Sichuan Province Emeishan City	9/12/2016	475	29°30′50″N 103°27′12″E	-	Meng Ye
92	*Z.armatum* 'Tengjiao'(EM)-4	ZA-TJ(EM)-4	Sichuan Province Emeishan City	9/12/2016	475	29°30′55″N 103°28′09″E	-	Meng Ye
93	*Z.armatum* 'Tengjiao'(EM)-5	ZA-TJ(EM)-5	Sichuan Province Emeishan City	9/12/2016	475	29°30′55″N 103°28′09″E	-	Meng Ye
94	*Z.armatum* 'Tengjiao'(EM)-6	ZA-TJ(EM)-6	Sichuan Province Emeishan City	9/12/2016	453	29°30′55″N 103°28′09″E	-	Meng Ye
95	*Z.armatum* 'Tengjiao'(EM)-7	ZA-TJ(EM)-7	Sichuan Province Emeishan City	9/12/2016	453	29°30′55″N 103°28′19″E	-	Meng Ye
96	*Z.armatum* 'Tengjiao'(EM)-8	ZA-TJ(EM)-8	Sichuan Province Emeishan City	9/12/2016	453	29°30′55″N 103°28′19″E	-	Meng Ye
97	*Z.armatum* 'Tengjiao'(EM)-9	ZA-TJ(EM)-9	Sichuan Province Emeishan City	9/12/2016	475	29°30′55″N 103°28′19″E	-	Meng Ye
98	*Z.armatum*-1 'Tengjiao' (Stingless, DL)	ZA-TJ(Sti,DL)-1	Sichuan Province Danling City	9/12/2016	475	29°59′26″N 103°24′57″E	stingless	Meng Ye
99	*Z.armatum* 'Tengjiao' (Stingless, DL)-2	ZA-TJ(Sti,DL)-2	Sichuan Province Danling City	9/12/2016	475	29°59′26″N 103°24′57″E	stingless	Meng Ye

100	*Z.armatum* ‘Tengjiao’(Stingless, DL)-3	ZA-TJ(Sti,DL)-3	Sichuan Province Danling City	9/12/2016	475	29°59′26″N 103°24′57″E	stingless	Meng Ye
101	*Z.armatum* ‘Tengjiao’(DL)-1	ZA-TJ(DL)-1	Sichuan Province Danling City	9/12/2016	475	29°59′26″N 103°24′57″E	-	Meng Ye
102	*Z.armatum* ‘Tengjiao’(DL)-2	ZA-TJ(DL)-2	Sichuan Province Danling City	9/12/2016	475	29°59′26″N 103°24′57″E	-	Meng Ye
103	*Z.armatum* ‘Tengjiao’(DL)-3	ZA-TJ(DL)-3	Sichuan Province Danling City	9/12/2016	467	29°59′26″N 103°24′57″E	-	Meng Ye
104	*Z.armatum* ‘Tengjiao’(DL)-GS	ZA-TJ(DL)-GS	Sichuan Province Danling County	-	1371	-	-	Meng Ye
105	*Z.armatum* ‘Jinyang Qinghuajiao’(DX)	ZA-JYQHJ(DX)	Sichuan Province Jinyang County	-	-	-	-	Meng Ye
106	*Z.armatum* ‘JinyangQinghuajiao’ (TP)	ZA-JYQHJ(TP)	SichuanProvince Jinyang County	-	1500	27°39'N 103°15'E	-	Meng Ye
107	*Z.armatum* ‘Jinyang Qinghuajiao’(MY)	ZA-JYQHJ(MY)	Sichuan Province Jinyang County	-	-	-	-	Meng Ye
108	*Z.armatum* ‘Jinyang Qinghuajiao’-1	ZA-JYQHJ-1	Sichuan Province Jinyang City	9/12/2016	467	27°34’N 103°9'E	-	Meng Ye
109	*Z.armatum* ‘Jinyang Qinghuajiao’-2	ZA-JYQHJ-2	Sichuan Province Jinyang City	9/12/2016	467	27°28’N 103°3'E	-	Meng Ye
110	*Z.armatum* ‘Jinyang Qinghuajiao’-3	ZA-JYQHJ-3	Sichuan Province Jinyang City	9/12/2016	472	27°26N 103°7’E	-	Meng Ye

111	*Z.armatum* 'Jinyang Qinghuajiao'-4	ZA-JYQHJ-4	Sichuan Province Jinyang City	9/12/2016	472	27°33'N 103°11'E	-	Meng Ye
112	*Z.armatum*'Qinghuajiao'	ZA-QHJ	Sichuan Province Dechang County	1/12/2016	-	-	-	Meng Ye
113	*Z.armatum* 'Jinyang Qinghuajiao'(XD)	ZA-JYQHJ(XD)	Sichuan Province Xide County	-	1600	-	-	Meng Ye
114	*Z.armatum* 'Zhaotong Qinghuajiao'	ZA-ZTQHJ	Yunnan Province Zhaotong City	2015	-	-	-	Meng Ye
115	*Z. piperitum* 'Chaocang Shanjiao'-1	ZP-CCSJ-1	Gansu Province Longnan City	30/6/2017	1354	33°27'46″N 104°57'102″	-	Meng Ye
116	*Z. piperitum* 'Chaocang Shanjiao'-2	ZP-CCSJ-2	Gansu Province Longnan City	30/6/2017	1355	33°27'46″N 104°57'102″	-	Meng Ye
117	*Z. piperitum* 'Chaocang Shanjiao'-3	ZP-CCSJ-3	Gansu Province Longnan City	30/6/2017	1356	33°27'46″N 104°57'102″	-	Meng Ye
118	*Z. piperitum* 'Liujin Shanjiao'-1	ZP-LJSJ-1	Gansu Province Longnan City	30/6/2017	1368	33°27'46″N 104°57'116″	-	Meng Ye
119	*Z. piperitum* 'Liujin Shanjiao'-2	ZP-LJSJ-2	Gansu Province Longnan City	30/6/2017	1369	33°27'46″N 104°57'116″	-	Meng Ye
120	*Z. piperitum* 'Liujin Shanjiao'-3	ZP-LJSJ-3	Gansu Province Longnan City	30/6/2017	1370	33°27'46″N 104°57'116″	-	Meng Ye
121	*Z. piperitum* 'Putao Shanjiao'	ZP-PTSJ	Gansu Province Longnan City	30/6/2017	1364	33°27'46″N 104°57'112″	-	Meng Ye
