# Supplementary material for: Six Express Sequence Tag–Simple Sequence Repeat Primers Reveal Genetic Diversity in the Cultivars of Three Zanthoxylum Species
Source: Curr Issues Mol Biol. 2023 Aug 30;45(9):7183–96. doi: 10.3390/cimb45090454 (PMC10529843; doi:10.3390/cimb45090454)
Supplement: Supplementary file 1 [file cimb-45-00454-s001.zip › Supplementary Table S2. Table information of six pairs of EST-SSR primers and genetic diversity results.pdf]

Supplementary Table S2. Table information of six pairs of EST-SSR primers and polymorphsim results

Accession	SSR sequence	Sequence of primer(5'-3')	Observed number of alleles (*Na*)	Effective number of alleles (*Ne*)	Nei's gene diversity (*H*)	Shannon's Information index (*I*)	polymorphic Loci
ZB23	$(CCAC)_5(CA)_8$	F:GGAGCTTCAGACTTGGTGCA<br>R:ACCAGGATCAGGTTCCACGA	2	1.1559	0.1086	0.1866	13
ZB26	$(AGG)_5$	F:ATTCACATCCACTGCGAGCA<br>R:GCCTTGCTGCTCTCCTTACA	2	1.3371	0.2164	0.3443	7
ZB28	(CTT)8	F:TGATAGCCACAGCCACAGTG<br>R:ACAGTGTGTGCCATCCTCAG	2	1.1969	0.1498	0.2612	5
ZB53	$(GCAACA)_7$	F:GCACGGCAACAGTTCGTATC<br>R:CCTGCCCCATTGAATTCCCT	2	1.5337	0.3205	0.4884	6
ZA26	$(GGA)_5(GGT)_5$	F:GCAGAACGATGAAGGGTGGA<br>R:TGAACCACAAACCACCACCA	2	1.2738	0.1608	0.2543	13
ZA51	$(TCCAAG)_5$	F:GGTGCCATGTCACAGGATCA<br>R:GTTTGTGACTGCTGCAAGGG	2	1.4961	0.2855	0.4287	7
