# Supplementary material for: Six Express Sequence Tag–Simple Sequence Repeat Primers Reveal Genetic Diversity in the Cultivars of Three Zanthoxylum Species
Source: Curr Issues Mol Biol. 2023 Aug 30;45(9):7183–96. doi: 10.3390/cimb45090454 (PMC10529843; doi:10.3390/cimb45090454)
Supplement: Supplementary file 1 [file cimb-45-00454-s001.zip › Supplementary Table S3. Table of Morphology characteristics of Z. bungeanum, Z. armatum and Z. piperitum..pdf]

Supplementary Table S3. Table of Morphology characteristics of *Z. bungeanum*, *Z. armatum* and *Z. piperitum*.

**Species**	***Z.bungeanum***	***Z.armatum***	***Z.piperitum***
Type and height	Deciduous shrub or tree<br>height 3-7m	Evergreen shrub or tree<br>height 3-5m	Deciduous shrub<br>height 3-5m
Prickle distribution	Stem and branch	Stem and branch	Stingless
Leaf	Oppositifolious,5-13 leaflets<br>oval or lanceolate<br>obsolete leaf wing<br>serrated leaf margin	Oppositifolious,3-9 leaflets<br>lanceolate usually<br>apparent leaf wing<br>obsolete serrated leaf margin	Oppositifolious,11-17 leaflets<br>Lanceolate<br>Leaf margins obtuse serrate
Flower	Unisexual flower<br>panicle<br>florescence:March to May	Unisexual flower,panicle<br>florescence:April to May	Unisexual flower,<br>Cymose panicles terminal<br>florescence:April
Fruit	Red fruit for mature<br>claret-colored fruit for mature<br>fructescence: August	Green fruit for immature<br>Red fruit for mature<br>fructescence: September	Red fruit for mature<br>fructescence: September to October
Flavour	Apparent aroma and spicy	Aromatic and pungent	Intensively aroma
